# Supplementary material for: Resolving the full spectrum of human genome variation using Linked-Reads
Source: Genome Res. 2019 Apr;29(4):635–45. doi: 10.1101/gr.234443.118 (PMC6442396; doi:10.1101/gr.234443.118)
Supplement: Supplemental Material [file supp_gr.234443.118_Supplemental_longranger-2.2.zip › longranger-2.2.tar.bz2!/lr2.2-public/lib/stan/cmdstan/stan/doc/api/html/agrad_2rev_2matrix_2trace__inv__quad__form__ldlt_8hpp_source.html]

Stan: src/stan/agrad/rev/matrix/trace\_inv\_quad\_form\_ldlt.hpp Source File


|  |  |
| --- | --- |
|  | Stan  2.5.0  probability, sampling & optimization |


- Main Page
- Related Pages
- Namespaces
- Classes
- Files

- File List
- File Members

All Classes Namespaces Files Functions Variables Typedefs Enumerations Enumerator Friends Macros Pages

- src
- stan
- agrad
- rev
- matrix

trace\_inv\_quad\_form\_ldlt.hpp

Go to the documentation of this file.

1 #ifndef STAN\_\_AGRAD\_\_REV\_\_MATRIX\_\_TRACE\_INV\_QUAD\_FORM\_LDLT\_HPP

2 #define STAN\_\_AGRAD\_\_REV\_\_MATRIX\_\_TRACE\_INV\_QUAD\_FORM\_LDLT\_HPP

3

4 #include <stan/math/matrix/Eigen.hpp>

5 #include <stan/agrad/rev/var.hpp>

6 #include <stan/agrad/rev/matrix/LDLT\_alloc.hpp>

7 #include <stan/agrad/rev/matrix/LDLT\_factor.hpp>

8 #include <boost/type\_traits/is\_same.hpp>

9 #include <boost/utility/enable\_if.hpp>

10 #include <stan/math/error\_handling/matrix/check\_multiplicable.hpp>

11

12 namespace stan {

13  namespace agrad {

14  namespace {

15  template <typename T2, int R2,int C2,typename T3,int R3,int C3>

16  class trace\_inv\_quad\_form\_ldlt\_impl : public chainable\_alloc {

17  protected:

18  inline void initializeB(const Eigen::Matrix<var,R3,C3> &B,bool haveD) {

19  Eigen::Matrix<double,R3,C3> Bd(B.rows(),B.cols());

20  \_variB.resize(B.rows(),B.cols());

21  for (int j = 0; j < B.cols(); j++) {

22  for (int i = 0; i < B.rows(); i++) {

23  \_variB(i,j) = B(i,j).vi\_;

24  Bd(i,j) = B(i,j).val();

25  }

26  }

27  AinvB\_ = \_ldlt.solve(Bd);

28  if (haveD)

29  C\_.noalias() = Bd.transpose()\*AinvB\_;

30  else

31  \_value = (Bd.transpose()\*AinvB\_).trace();

32  }

33  inline void initializeB(const Eigen::Matrix<double,R3,C3> &B,bool haveD) {

34  AinvB\_ = \_ldlt.solve(B);

35  if (haveD)

36  C\_.noalias() = B.transpose()\*AinvB\_;

37  else

38  \_value = (B.transpose()\*AinvB\_).trace();

39  }

40

41  template<int R1,int C1>

42  inline void initializeD(const Eigen::Matrix<var,R1,C1> &D) {

43  D\_.resize(D.rows(),D.cols());

44  \_variD.resize(D.rows(),D.cols());

45  for (int j = 0; j < D.cols(); j++) {

46  for (int i = 0; i < D.rows(); i++) {

47  \_variD(i,j) = D(i,j).vi\_;

48  D\_(i,j) = D(i,j).val();

49  }

50  }

51  }

52  template<int R1,int C1>

53  inline void initializeD(const Eigen::Matrix<double,R1,C1> &D) {

54  D\_ = D;

55  }

56

57  public:

58  template<typename T1, int R1,int C1>

59  trace\_inv\_quad\_form\_ldlt\_impl(const Eigen::Matrix<T1,R1,C1> &D,

60  const stan::math::LDLT\_factor<T2,R2,C2> &A,

61  const Eigen::Matrix<T3,R3,C3> &B)

62  : Dtype\_(boost::is\_same<T1,var>::value?1:0),

63  \_ldlt(A)

64  {

65  initializeB(B,true);

66  initializeD(D);

67

68  \_value = (D\_\*C\_).trace();

69  }

70

71  trace\_inv\_quad\_form\_ldlt\_impl(const stan::math::LDLT\_factor<T2,R2,C2> &A,

72  const Eigen::Matrix<T3,R3,C3> &B)

73  : Dtype\_(2),

74  \_ldlt(A)

75  {

76  initializeB(B,false);

77  }

78

79  const int Dtype\_; // 0 = double, 1 = var, 2 = missing

80  stan::math::LDLT\_factor<T2,R2,C2> \_ldlt;

81  Eigen::Matrix<double,Eigen::Dynamic,Eigen::Dynamic> D\_;

82  Eigen::Matrix<vari\*,Eigen::Dynamic,Eigen::Dynamic> \_variD;

83  Eigen::Matrix<vari\*,R3,C3> \_variB;

84  Eigen::Matrix<double,R3,C3> AinvB\_;

85  Eigen::Matrix<double,C3,C3> C\_;

86  double \_value;

87  };

88

89  template <typename T2,int R2,int C2,typename T3,int R3,int C3>

90  class trace\_inv\_quad\_form\_ldlt\_vari : public vari {

91  protected:

92  static inline void chainA(const double &adj,

93  trace\_inv\_quad\_form\_ldlt\_impl<double,R2,C2,T3,R3,C3> \*impl) {

94  }

95  static inline void chainB(const double &adj,

96  trace\_inv\_quad\_form\_ldlt\_impl<T2,R2,C2,double,R3,C3> \*impl) {

97  }

98

99  static inline void chainA(const double &adj,

100  trace\_inv\_quad\_form\_ldlt\_impl<var,R2,C2,T3,R3,C3> \*impl) {

101  Eigen::Matrix<double,R2,C2> aA;

102

103  if (impl->Dtype\_ != 2)

104  aA.noalias() = -adj\*(impl->AinvB\_ \* impl->D\_.transpose() \* impl->AinvB\_.transpose());

105  else

106  aA.noalias() = -adj\*(impl->AinvB\_ \* impl->AinvB\_.transpose());

107

108  for (int j = 0; j < aA.cols(); j++)

109  for (int i = 0; i < aA.rows(); i++)

110  impl->\_ldlt.\_alloc->\_variA(i,j)->adj\_ += aA(i,j);

111  }

112  static inline void chainB(const double &adj,

113  trace\_inv\_quad\_form\_ldlt\_impl<T2,R2,C2,var,R3,C3> \*impl) {

114  Eigen::Matrix<double,R3,C3> aB;

115

116  if (impl->Dtype\_ != 2)

117  aB.noalias() = adj\*impl->AinvB\_\*(impl->D\_ + impl->D\_.transpose());

118  else

119  aB.noalias() = 2\*adj\*impl->AinvB\_;

120

121  for (int j = 0; j < aB.cols(); j++)

122  for (int i = 0; i < aB.rows(); i++)

123  impl->\_variB(i,j)->adj\_ += aB(i,j);

124  }

125

126  public:

127  trace\_inv\_quad\_form\_ldlt\_vari(trace\_inv\_quad\_form\_ldlt\_impl<T2,R2,C2,T3,R3,C3> \*impl)

128  : vari(impl->\_value), \_impl(impl)

129  {}

130

131  virtual void chain() {

132  // F = trace(D \* B' \* inv(A) \* B)

133  // aA = -aF \* inv(A') \* B \* D' \* B' \* inv(A')

134  // aB = aF\*(inv(A) \* B \* D + inv(A') \* B \* D')

135  // aD = aF\*(B' \* inv(A) \* B)

136  chainA(adj\_, \_impl);

137

138  chainB(adj\_, \_impl);

139

140  if (\_impl->Dtype\_ == 1) {

141  for (int j = 0; j < \_impl->\_variD.cols(); j++)

142  for (int i = 0; i < \_impl->\_variD.rows(); i++)

143  \_impl->\_variD(i,j)->adj\_ += adj\_\*\_impl->C\_(i,j);

144  }

145  }

146

147  trace\_inv\_quad\_form\_ldlt\_impl<T2,R2,C2,T3,R3,C3> \*\_impl;

148  };

149

150  }

151

152

158  template <typename T2,int R2,int C2,typename T3,int R3,int C3>

159  inline typename

160  boost::enable\_if\_c<boost::is\_same<T2,var>::value ||

161  boost::is\_same<T3,var>::value, var>::type

162  trace\_inv\_quad\_form\_ldlt(const stan::math::LDLT\_factor<T2,R2,C2> &A,

163  const Eigen::Matrix<T3,R3,C3> &B)

164  {

165  stan::math::check\_multiplicable("trace\_inv\_quad\_form\_ldlt(%1%)",A,"A",

166  B,"B",(double\*)0);

167

168  trace\_inv\_quad\_form\_ldlt\_impl<T2,R2,C2,T3,R3,C3> \*\_impl = new trace\_inv\_quad\_form\_ldlt\_impl<T2,R2,C2,T3,R3,C3>(A,B);

169

170  return var(new trace\_inv\_quad\_form\_ldlt\_vari<T2,R2,C2,T3,R3,C3>(\_impl));

171  }

172

173  }

174 }

175

176 #endif

LDLT\_alloc.hpp

check\_multiplicable.hpp

Dtype\_

const int Dtype\_

**Definition:** trace\_inv\_quad\_form\_ldlt.hpp:79

\_impl

trace\_inv\_quad\_form\_ldlt\_impl< T2, R2, C2, T3, R3, C3 > \* \_impl

**Definition:** trace\_inv\_quad\_form\_ldlt.hpp:147

\_variD

Eigen::Matrix< vari \*, Eigen::Dynamic, Eigen::Dynamic > \_variD

**Definition:** trace\_inv\_quad\_form\_ldlt.hpp:82

stan::math::check\_multiplicable

bool check\_multiplicable(const char \*function, const T1 &y1, const char \*name1, const T2 &y2, const char \*name2, T\_result \*result)

**Definition:** check\_multiplicable.hpp:15

AinvB\_

Eigen::Matrix< double, R3, C3 > AinvB\_

**Definition:** trace\_inv\_quad\_form\_ldlt.hpp:84

\_variB

Eigen::Matrix< vari \*, R3, C3 > \_variB

**Definition:** trace\_inv\_quad\_form\_ldlt.hpp:83

stan::math::LDLT\_factor

**Definition:** LDLT\_factor.hpp:16

\_value

double \_value

**Definition:** trace\_inv\_quad\_form\_ldlt.hpp:86

var.hpp

Eigen.hpp

stan::agrad::var

Independent (input) and dependent (output) variables for gradients.

**Definition:** var.hpp:27

D\_

Eigen::Matrix< double, Eigen::Dynamic, Eigen::Dynamic > D\_

**Definition:** trace\_inv\_quad\_form\_ldlt.hpp:81

\_ldlt

stan::math::LDLT\_factor< T2, R2, C2 > \_ldlt

**Definition:** trace\_inv\_quad\_form\_ldlt.hpp:80

stan::math::trace

T trace(const Eigen::Matrix< T, Eigen::Dynamic, Eigen::Dynamic > &m)

Returns the trace of the specified matrix.

**Definition:** trace.hpp:20

C\_

Eigen::Matrix< double, C3, C3 > C\_

**Definition:** trace\_inv\_quad\_form\_ldlt.hpp:85

stan::agrad::trace\_inv\_quad\_form\_ldlt

boost::enable\_if\_c< boost::is\_same< T2, var >::value||boost::is\_same< T3, var >::value, var >::type trace\_inv\_quad\_form\_ldlt(const stan::math::LDLT\_factor< T2, R2, C2 > &A, const Eigen::Matrix< T3, R3, C3 > &B)

Compute the trace of an inverse quadratic form.

**Definition:** trace\_inv\_quad\_form\_ldlt.hpp:162

LDLT\_factor.hpp


---

[ Stan Home Page ]

*© 2011–2014,
Stan Development Team.*
